# End-to-End Depth-Guided Relighting Using Lightweight Deep Learning-Based Method

**DOI:** 10.3390/jimaging9090175

**Published:** 2023-08-28

**Authors:** Sabari Nathan, Priya Kansal

**Affiliations:** Couger Inc., Tokyo 150-0001, Japan

**Keywords:** image enhancement, image relighting, depth-guided

## Abstract

Image relighting, which involves modifying the lighting conditions while preserving the visual content, is fundamental to computer vision. This study introduced a bi-modal lightweight deep learning model for depth-guided relighting. The model utilizes the Res2Net Squeezed block’s ability to capture long-range dependencies and to enhance feature representation for both the input image and its corresponding depth map. The proposed model adopts an encoder–decoder structure with Res2Net Squeezed blocks integrated at each stage of encoding and decoding. The model was trained and evaluated on the VIDIT dataset, which consists of 300 triplets of images. Each triplet contains the input image, its corresponding depth map, and the relit image under diverse lighting conditions, such as different illuminant angles and color temperatures. The enhanced feature representation and improved information flow within the Res2Net Squeezed blocks enable the model to handle complex lighting variations and generate realistic relit images. The experimental results demonstrated the proposed approach’s effectiveness in relighting accuracy, measured by metrics such as the PSNR, SSIM, and visual quality.

## 1. Introduction

The task of relighting involves the transfer of the color temperature and the light source direction from one illumination setting to another illumination setting. The setting may either be user-defined or random. Despite the complexity involved in this task, there are many implications, especially with the latest developments in technologies and their applications. For example, with the recent innovations in digital and smartphone cameras, the illumination in the images is controlled in real-time. However, maintaining the illumination setting mitigates the outdoor lighting effect, the weather conditions, the shadows of nearby objects, sunlight reflections, etc. The digital cameras available on the market offer techniques to remove unwanted lighting effects or add the required lighting effects. Still, these techniques require much human expertise and intervention. Despite this, in the age of augmented reality (AR) devices, different illumination settings, for example, the distant light sourcesin both outdoor and indoor scenes, are greatly needed to enhance the user experience in many domains, especially online shopping for interior furnishings. Image relighting also has significant implications for the gaming industry, wherein scene relighting is performed based on user instructions. Similarly, we cannot deny the utility of image-relighting tasks in forensic analysis and surveillance applications. In forensic analysis and surveillance applications, one-to-one relighting can enhance the details in images captured under challenging lighting conditions. This can help investigators and law enforcement agencies gain better insights regarding the evidence and scenes. Besides medical image analysis, computer graphics and animation, artistic expression, and visual effects, image relighting helps create better results. Recently, researchers have been exploring the applications for training data augmentation by generating variations of images with different lighting conditions; models can be trained to be more-robust and -generalizable to various lighting scenarios.

**Figure 1:**
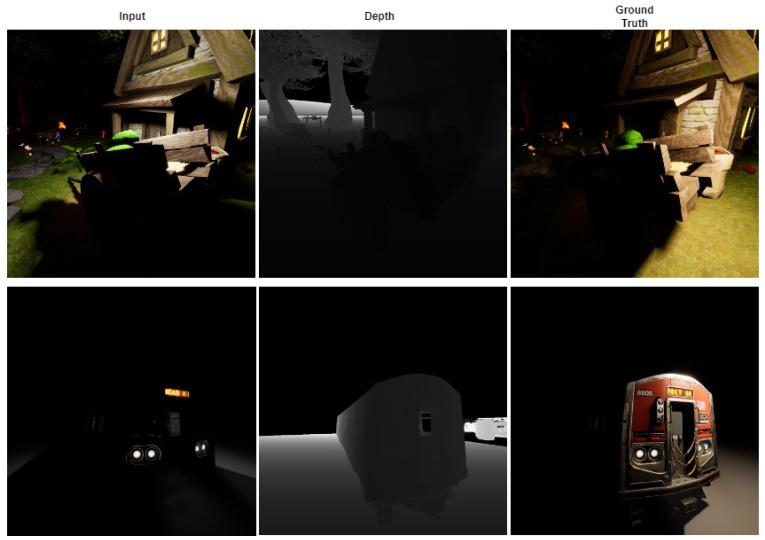
Example images from the VIDIT dataset [6].

To manage the illumination settings, many techniques such as histogram equalization, gamma correction, retinex-theory-based methods [1,2], low-light image-enhancement methods [3], shadow removal [4,5], etc., are available. However, these techniques focus on correcting the existing lighting conditions, rather than changing the current ones to different lighting conditions. Transferring the images from the input lighting settings to predefined lighting settings is a challenging task. This task involves the constraints of existing shadows, different light source directions, existing unlit sources, etc. Some of the examples from the VIDIT dataset [6] are presented in Figure 1. Deep learning methods have proven their effectiveness in almost all the operations related to image-to-image-translation. The benefits of using deep learning methods are that these techniques do not require any prior information such as geometric priors, etc. Moreover, it is easy to generalize the results, and no human intervention is necessary once the model has been trained. Recently, some deep-learning-based methods [1,7,8,9,10,11] have been proposed without explicit inverse rendering steps for estimating the scene properties.

However, deep-learning-based methods suffer from substantial data requirements or complex and high-cost computations, making them unsuitable for real-time applications. Hence, there is a need for a new architecture that can achieve state-of-the-art results even when trained on fewer images and that can effectively relight the images in real-time. The proposed model tackles this problem using a bimodal encoder–decoder structure with Res2Net Squeezed blocks, inspired by [12], integrated into each stage. The encoder module separately captures multi-scale features from the input and corresponding depth images. In contrast, the decoder module reconstructs the relit image by progressively upsampling and merging the RGB and depth features. The Res2Net Squeezed blocks within each module provide increased receptive fields, allowing the model to capture fine-grained details and high-level semantic information without any added computation. The proposed model achieves state-of-the-art performance with only 454,159 parameters and works in real-time. In short, the contributions of the proposed model are as follows:

- A new U-Net-based architecture characterized by fewer parameters is proposed. To our knowledge, this is the smallest-size model among all existing methods for image relighting that can achieve competitive performance.
- A modified version of the Res2Net blocks, known as Res2Net Squeezed blocks, which implicitly extends the receptive field area and thus collects and retains more information about the image, is proposed.
- A depth-guided stream is introduced, which is merged with the corresponding RGB features of the same size and then progressively up-sampled to obtain the target images.
- A bi-modal depth-guided model that extracts the features from the depth and RGB images using two streams is designed. This model implicitly enhances the receptive field by utilizing the Res2Net Squeezed blocks for image relighting. Extensive experiments and a comparative analysis demonstrated that our proposed method outperformed the others while maintaining high computational efficiency.

## 2. Related Work

This section provides a concise overview of existing studies in the same context. Manipulating image lighting conditions is a complex task that demands meticulous execution. Traditionally, the practice of inverse rendering [13] has been employed to facilitate modifying illumination conditions. Techniques such as using specialized optical equipment to assess geometry [14,15,16], evaluating surface reflectance [7,17], analyzing environmental illumination [18,19], and reversing global illumination within the scene [20] have gained widespread popularity as fundamental approaches for inverse rendering. However, it is important to acknowledge that these methods are inherently complex, resource-intensive, and require significant manual intervention from domain experts.

Furthermore, image-based techniques such as histogram equalization, gamma correction, and solutions based on the retinex theory [1,2] are extensively used for illumination estimation and manipulation. Nevertheless, these methodologies have certain limitations, primarily in generating only rudimentary, low-level manipulations that are often vague and ambiguous.

To address the challenge of producing ambiguous outcomes, image relighting, illumination manipulation, and illumination estimation have shifted towards learning-based methods [21,22]. Deep learning neural networks, in particular, have emerged as powerful tools, demonstrating remarkable capabilities in various image enhancement tasks. These tasks encompass a diverse range of subtasks, including colorizing black and white images [23], restoring damaged images [24,25], removing moiré patterns from images [26], denoising noisy images [27], deblurring blurred images [28], and enhancing image resolution [29], among others.

In this context, it is crucial to highlight that image illumination manipulation comprises various subtasks. Some of the widely addressed challenges include existing illumination correction [30,31,32], shadow removal [33], estimation of illumination effects [34], determination of illumination direction [34], and the actual process of relighting images [8,9,34].

However, our present paper focuses on the specific problem of one-to-one image relighting. This involves predicting an output image with a target illumination setting based on an input image characterized by diverse and unknown illumination angles and color temperature. Our research efforts are primarily centered around the VIDIT dataset, which was ingeniously proposed by Helou et al. [6] and subsequently used in illumination transfer and image relighting competitions, namely, AIM 2020 [8] and NTIRE 2021 [9]. These competitions have witnessed the participation of various researchers, leading to the development of winning solutions that incorporated and adapted existing modules or networks, which have previously demonstrated impressive representation capabilities in other domains.

To delve deeper into some noteworthy examples, the winners of AIM 2020, Puthessery et al., devised WDRN [1], a novel approach leveraging wavelet transformations to enable efficient multi-scale representations. Additionally, Paul et al. [35] skillfully integrated pix2pix [36] into their framework, harnessing the power of adversarial learning to enhance the quality of their generated images further. Moreover, Yang et al. [37] ingeniously integrated depth maps into their relighting network, relying on an RGB-D saliency detection method to guide their depth-guided relighting process. In another notable work, S3net [10], the authors ingeniously combined RGB images with depth images during the feature extraction stage.

Inspired by the winning solution [38], our proposed model utilizes two separate streams in the encoder to estimate features from the RGB image and depth maps. Unlike MBNet [38], our innovation lies in fusing the depth and RGB features together using an attention mechanism within the decoder module before upsampling. The subsequent section will delve into the details of our proposed architecture, highlighting its unique features and improvements over existing methodologies.

## 3. Approach and Proposed Network

### 3.1. Task Definition

In a one-to-one relighting task, the input and the target lighting settings are pre-determined and fixed for all the captured scenes. We have formulated this problem as

(1) $$I_{out}=f\left(I_{in},{Depth}_{in}\right),$$

where $I_{out}$ is the target relit image, and $I_{in}$ and ${Depth}_{in}$ represent the RGB input image and depth image, respectively. *f* represents the model we designed to obtain the relighted images. This is similar to almost all image-to-image-translation tasks; however, here, we are adding the corresponding depth maps as the guide.

### 3.2. Detail of the Architecture

**Figure 2:**
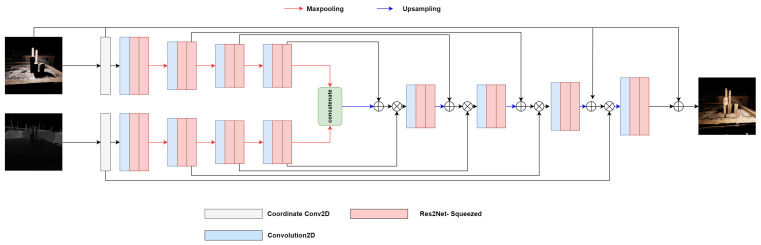
A detailed view of the proposed architecture. The proposed architecture consists of a two-stream encoder for RGB images and depth input, respectively. The block outputs of the RGB stream are concatenated with the decoder’s output using the skip connections, and the block outputs of the depth stream are multiplied using element-wise multiplication. The network is trained end-to-end.

The proposed network architecture is designed to extract depth effectively and image features through two encoder structures. The details of the architecture are illustrated in Figure 2. The image and depth image each go through the coordinate convolution layer, allowing the capture of spatial patterns and correlations specific to each modality. The resulting feature maps from these blocks are further processed by their corresponding encoder blocks. Each encoder block consists of two Res2Net Squeezed blocks [12,39], followed by down-sampling layers that progressively reduce the spatial dimensions. This hierarchical representation enables the extracting of local and global contextual information from the input data. Specifically, the image encoder path comprises four encoder blocks, while the depth encoder path also consists of four encoder blocks, ensuring comprehensive feature extraction from both modalities.

To fuse the extracted features and generate the final output, the outputs of the two encoder blocks are concatenated and passed to the decoder block. The decoder block reconstructs the output by gradually increasing the size of the feature maps through upsampling layers. Similar to the U-net [39,40,41] architecture, the decoder employs skip connections to concatenate feature maps of the same size from earlier layers, facilitating the integration of both low-level and high-level information. This process enhances the network’s capacity to recover detailed and contextual features in the relit images. Notably, a significant departure from conventional approaches is that our network is trained to learn the residual information instead of directly predicting the full images. As a result, the final output is obtained by taking the difference between the original and relit images. This strategy of residual learning enables the network to focus on capturing and reconstructing the variations or changes induced by the relighting process. This approach promotes more efficient training and empowers the generation of high-quality relit images with enhanced fine details and subtle variations.

In summary, the proposed network architecture effectively integrates depth and image features through separate encoding paths, leveraging the power of Res2Net Squeezed blocks and skipping connections in the decoding stage. Training the network to learn the residual information allows for accurately capturing variations between the original and relit images. This results in superior relighting performance and a finer level of control in the relighting process.

### 3.3. Coordinate Convolution Layer

**Figure 3:**
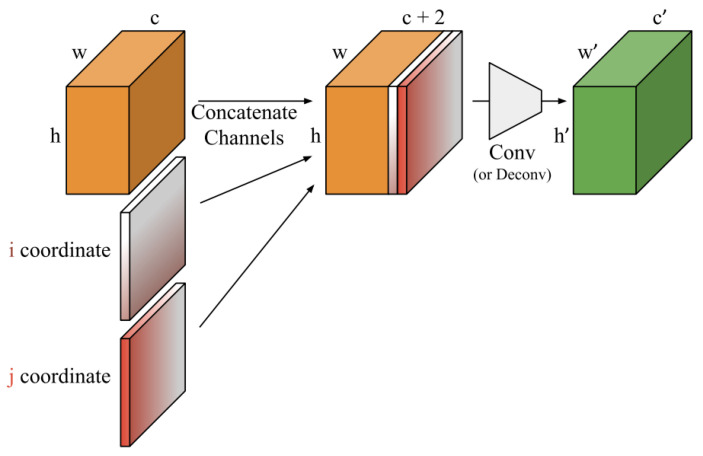
Coordinate convolutional layer as proposed in the original paper [42].

The 2D coordinate convolution layer [41,42] is a fundamental component of convolutional neural networks (CNNs) that operates on 2D spatial data, such as images. Figure 3 shows the detailed coordinate convolutional layer as given in the original paper. It performs convolution by combining the input feature map with a set of learnable filters. In this layer, each filter is associated with a specific 2D coordinate position, represented by its center. The output at each spatial location is computed by convolving the corresponding filter with the input feature map centered at that position, followed by a nonlinear activation function. Mathematically, the output feature map can be expressed as

(2) $$Y_{i,j}=\sum_{m=1}^{M}\sum_{n=1}^{N}W_{m,n}\cdot X_{i+m,j+n}+b,$$

where $Y_{i,j}$ denotes the output feature map at position (i,j), $X_{i+m,j+n}$ represents the input feature map at position (i+m,j+n), $W_{m,n}$ is the learnable filter associated with the coordinate offset (m,n), and b is a bias term. The resulting feature maps capture local spatial patterns, enabling the network to learn hierarchical representations of the input data.

### 3.4. Res2Net-Squeezed

**Figure 4:**
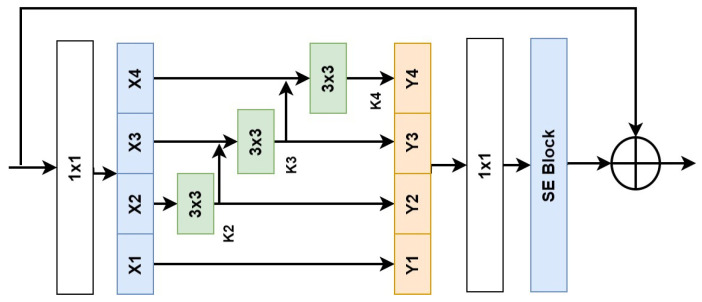
Block diagram of Res2Net-Squeezed block [12].

The Res2Net-Squeezed [12] block (Figure 4) is an extension of the Res2Net block, designed to enhance the representation power of deep neural networks. It introduces “squeezing” further to exploit the hierarchical features within a Res2Net block. In this block, the input feature map is divided into multiple branches, each processing a different scale of information. Convolutional layers with different dilation rates are applied within each branch to capture multi-scale context. Additionally, the LeakyReLU [41] activation function is incorporated within the convolutional block to introduce non-linearity. This modification helps alleviate the vanishing gradient problem by allowing a small negative slope for negative input values, thereby enhancing the learning capability of the network. The modified Res2Net-Squeezed block can be represented mathematically as

(3) $$Y=F\left(X\right)=X+\sum_{i=1}^{n}W_{i}*\mathrm{LeakyReLU}\left(X\right),$$

where Y denotes the output feature map, X represents the input feature map, $W_{i}$ represents the weights of the *i*-th convolutional layer, and ∗ denotes the convolution operation. The LeakyReLU activation function is applied element-wise within the convolutional block, ensuring that the gradients can flow backwards even for negative input values.

By incorporating the LeakyReLU [41] activation layer within the Res2Net-Squeezed block, the modified architecture benefits from the multi-scale context captured by the Res2Net mechanism and gains the ability to learn more expressive and robust representations by introducing non-linearity.

### 3.5. Squeeze-and-Excitation

The Squeezed block [43] is introduced to enhance further the selection of informative features within each scale of the Res2Net block. It is designed to capture the most relevant contextual information while suppressing less useful information. By doing so, the Squeezed block effectively promotes feature maps with high discriminative power, leading to more effective feature representation.

The core idea of the Squeezed block is to employ global pooling operations, such as global average pooling (GAP) or global max pooling (GMP), to reduce the spatial dimensions of each feature map. This pooling operation aggregates information across the entire spatial extent of the feature map, forcing the network to focus on the most salient and discriminative features.

After the global pooling operation, the reduced feature maps undergo a squeeze-and-excitation mechanism. The mechanism aims to recalibrate the channel-wise feature responses to highlight important channels and suppress less informative ones. This step is crucial in enhancing the feature selection process within each scale of the Res2Net block.

Let $X\in R^{H\times W\times C}$ be the input feature map. The Squeezed block operation is defined as follows:

(4) $$Z_{c}=\frac{1}{H\times W}\sum_{i=1}^{H}\sum_{j=1}^{W}X_{i,j,c},\;\forall c=1,2,\ldots ,C$$

(5) $$E_{c}=\sigma \left(FC_{2}\left(\delta \left(FC_{1}\left(Z_{c}\right)\right)\right)\right),\;\forall c=1,2,\ldots ,C$$

(6) $$Y_{i,j,c}=E_{c}\cdot X_{i,j,c},\;\forall i=1,2,\ldots ,H;j=1,2,\ldots ,W;c=1,2,\ldots ,C,$$

where $FC_{1}$ and $FC_{2}$ are fully connected layers, δ(·) is the ReLU activation function, and σ(·) is the sigmoid activation function.

The output *Y* represents the squeezed feature map, which is used in combination with the original Res2Net block features to create a more expressive and discriminative representation for downstream tasks.

## 4. Experimental Setup

### 4.1. Dataset

The novel VIDIT [6] dataset was employed for this study, comprising 300 training scenes, while the validation and test set each consisted of 90 scenes, with an equal distribution between them. Notably, the scenes in each set were mutually exclusive. Each scene was captured in the dataset 40 times, encompassing 8 equally-spaced azimuthal angles and five different color temperatures for the illumination. The images had a resolution of 1024 × 1024 and one sample consisted of input images, corresponding depth map and ground truth. Except for the ground-truth test data, the complete dataset is publicly available online [6]. In research papers outside of the challenge, it is customary for authors to present their results based on the validation set for reporting purposes. The same is followed for result reporting in this paper. However, for training purpose, the training data is split into an 80–20 ratio. Hence, out of 300 training images, 240 images are used for training and 60 images are used for validating the model.

### 4.2. Data Augmentation

Data augmentation is vital for training an image-relighting model to enhance its generalization and robustness. This augmentation process involves applying horizontal shifting, vertical shifting, and 90-degree rotation to input RGB images, depth images, and ground truth images. Horizontal and vertical shifting involves moving the images along the x- and y-axes, introducing diversity in object positions and perspectives. The 90-degree rotation augments the images by altering their orientation.

By applying these augmentations, the model can handle variations in lighting conditions and object placements that may occur in real-world scenarios. The increased variability in the training data improves the model’s ability to generalize across different lighting scenarios, making it more reliable and accurate when applied to relight images. Consequently, this data augmentation strategy empowers the image-relighting model to achieve better results and effectively adapt to various lighting conditions, enhancing its overall performance and applicability.

### 4.3. Loss Function

The model is trained using three distinct loss functions: mean absolute error (MAE) loss, structural similarity index (SSIM) loss, and gradient loss. Let *N* be the number of samples in each batch, and the average mean squared error loss is defined as follows:

(7) $$L_{MAE}=\frac{1}{N}\sum_{i=1}^{N}∥f\left(x_{i}\right)-x_{i})∥.$$

In this equation, $x_{i}$ represents the input degraded image and $f(x_{i})$ represents the restored image using our model.

Next, the SSIM loss is defined as

(8) $$L_{SSIM}=\frac{1}{N}\sum {i=1}^{N}\left(1-SSIM\left(x_{i},f\left(x_{i}\right)\right)\right),$$

where SSIM is the structural similarity index function as defined in the paper [44].

Lastly, the gradient loss [45] is computed as the $L_{1}$ distance between the gradients of *y* and $\hat{y}$:

(9) $$L_{Grad}=\frac{1}{N}\sum {i=1}^{N}\left|\nabla_{x}y-\nabla_{x}\hat{y}\right|1+{\left|\nabla yy-\nabla_{y}\hat{y}\right|}_{1}.$$

Therefore, the final loss function is a weighted sum of these three losses:

(10) $$T_{Loss}=0.15\cdot L_{SSIM}+L_{MAE}+L_{Grad},$$

where the coefficient 0.15 is used to adjust the importance of the SSIM loss compared to the other two losses.

### 4.4. Training

We normalized the images to a range of 0 to 1 during the training process. No additional pre- or post-processing steps were applied to ensure simplicity and efficiency during inference. To update the model weights, we utilized the Adam optimizer. The initial learning rate was set to 0.001, and if the validation loss did not improve after 15 epochs, the learning rate was reduced by 10%. With a batch size of 2, the model was evaluated using the peak-signal-to-noise ratio (PSNR) and structural similarity index (SSIM). Training took place for 200 epochs, utilizing a 16 GB NVIDIA Tesla K80 GPU on Google Colab pro.

### 4.5. Ablation Study

In this section, we present ablation studies that demonstrate the effectiveness of our method and provide detailed analyses of the proposed modules. The key components of our model are the coordinate convolution layer and the Res2Net-Squeezed block. Our primary focus is on examining the impact of these two components on the performance of our proposed network. To begin, we verify the effectiveness of the residual learning strategy when combined with its extended squeezed version. Following this, we discuss the influence of using the coordinate convolution layer. All ablation studies were conducted using the VIDIT [6] dataset.

#### 4.5.1. Experiments on Residual Strategy

The Res2Net-Squeezed block plays a crucial role in the proposed network, serving as a foundational component for both the encoder and decoder segments. By incorporating multi-scale feature fusion, the Res2Net-Squeezed block enables the capture and combine of features from multiple receptive fields. The efficacy of the Res2Net-Squeezed block is outlined in Table 1.

Table 1 illustrates the utilization of distinct block variations. The vanilla Res2Net configuration employs the original Res2Net block [12] as its core. The vanilla residual block [47] embodies the original residual block. In the absence of any variation of residual learning, the model is trained using a plain convolution block as well, denoted as “w/o residual learning”. Finally, the Res2Net-Squeezed block signifies the block integrated into the proposed model.

It is evident from Table 1 that the evaluation metrics decrease significantly without the residual learning strategy, indicating the importance of both residual mappings and identity mapping. However, the vanilla residual block alone is not sufficient. By employing squeezed attention in the Res2Net block, the model becomes capable of concentrating on more relevant channel features while discarding irrelevant features. This results in a highly efficient feature representation.

#### 4.5.2. Experiments on Coordinate Convolutional Layer

In tasks involving image-to-image translation, where information about the spatial relationships among different regions holds paramount importance, the inclusion of additional spatial information enhances the learning process. Table 2 illustrates the variations in performance when employing the coordinate convolutional layer and when omitting it.

The metrics presented in Table 2 highlight the importance of spatial awareness created by the coordinate convolutional layer. The coordinate convolutional layer facilitated the model in effectively learning geometric transformations under varying lighting conditions, particularly when dealing with varying shadow positions resulting from diverse lighting directions in input and target images.

### 4.6. Results and Comparison with State-of-the-Art Methods

#### 4.6.1. Comparison for Evaluation Metrics

This section provides a comparison between our method and other state-of-the-art (SOTA) relighting techniques. We primarily utilized the winning and runner-up solutions from both AIM 2020 [8] and AIM 2021 [9] challenges. This encompasses WDRN [1], which secured the top position in AIM 2020 [8], and DRN [48], which attained the best PSNR score in AIM 2020 [8]. We also consider MBNet [38], which claimed the first spot in AIM 2021 [9], and OIDDR-Net [49], the runner-up method from AIM 2021 [9]. Furthermore, we incorporate some of the latest state-of-the-art methods designed for the same task, including IAN [50] and S3Net [10], as well as typical image-to-image translation approaches such as pix2pix [36] and DPR [51]—a state-of-the-art portrait-relighting method—for further comparison. It is crucial to note that these results lack full reproducibility due to the absence of open-sourced code from multiple approaches. Consequently, the results are extracted from their published research papers.

To conduct a quantitative assessment, we employ the PSNR and SSIM [44] metrics, focusing on the RGB channels of the relit outcomes. Additionally, the evaluation incorporates the LPIPS metric [52], renowned for its strong alignment with human judgments. These assessments are carried out across the VIDIT dataset [6]. The outcomes are comprehensively summarized in Table 3, which provides a comparative overview of our model vis-à-vis the other techniques discussed earlier.

Table 3 shows that we achieve competitive results using models with fewer parameters than the other competitive methods.

#### 4.6.2. Comparison for Qualitative Results

**Figure 5:**
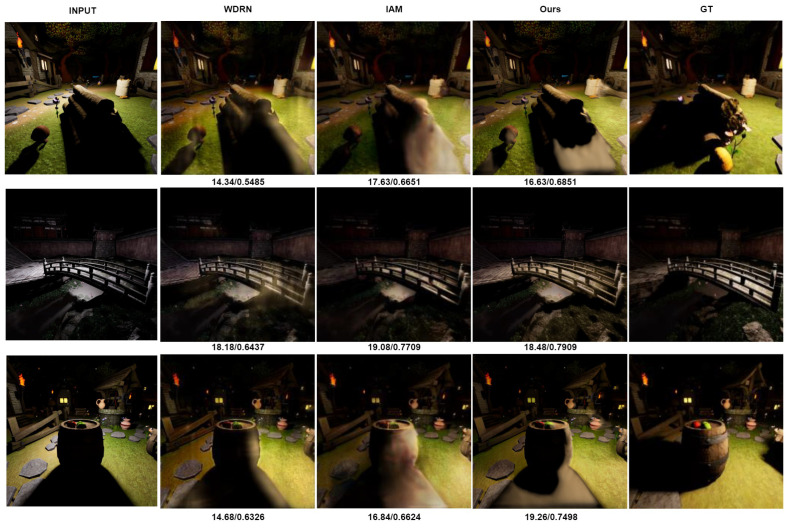
Qualitative comparison of resulting images (our method v.s. AIM 2020 winners and latest state-of art methods).

**Figure 6:**
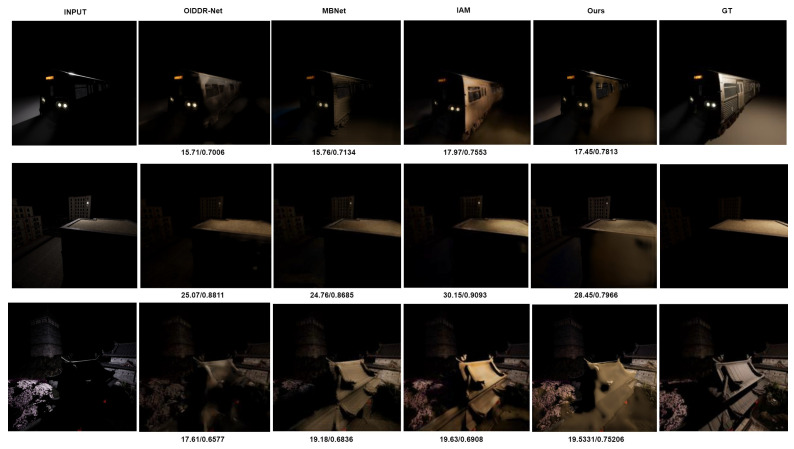
Qualitative comparison of resulting images (our method v.s. NITRE 2021 winners and latest state-of art methods).

In addition to the quantitative analysis, an assessment of the enduring quality of the generated images across various state-of-the-art methodologies is undertaken. Despite being relatively lightweight compared to the existing array of methods, the results underscored that the proposed model is capable of producing outputs of comparable or even superior quality. To facilitate visual comparison between the proposed model’s predictions and the actual ground truth images for each input sample, corresponding images are presented in Figure 5 and Figure 6. These figures showcase the input images, the resultant outputs, and their corresponding ground truth counterparts.

#### 4.6.3. Comparison for Model Size

**Figure 7:**
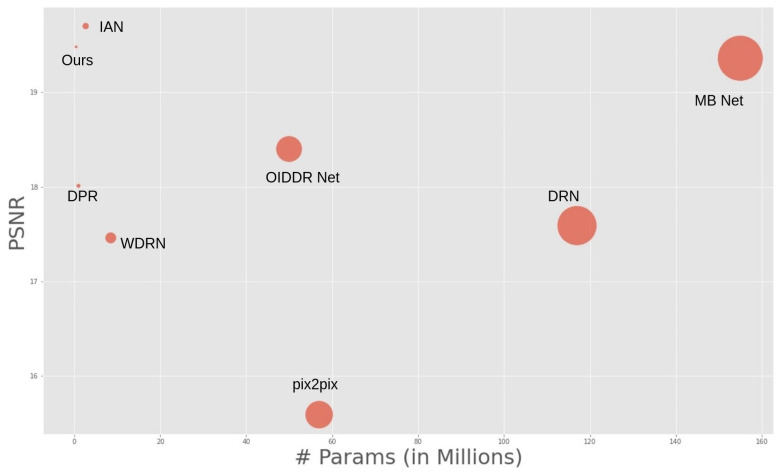
Comparison of parameters and performance of state-of-the art methods.

In terms of parameter count, the proposed approach showcased the lowest figures among all the prevailing methodologies. We present Figure 7 and Table 4 to provide a comprehensive overview of this parameter-performance relationship for the VIDIT dataset [6]. These visualizations highlight the dominance of our method in terms of performance and parameter efficiency. Our approach outperformed in delivering results of remarkable perceptual quality while maintaining a notably diminished parameter count compared to the state-of-the-art alternatives.

## 5. Discussion

The proposed network introduces a lightweight relighting model specifically designed to incorporate squeezed attention into the channel slices of the input. This strategic approach enhances the model’s efficiency in capturing both global and contextual features.

Within the context of our study, a comprehensive ablation study (conducted in Section 4.4) has effectively highlighted the crucial role played by each individual module that constitutes the core of our proposed model. Furthermore, the comparative analysis presented in Section 4.5 provides clear evidence of the competitive edge of our proposed method.

However, it is important to acknowledge that our proposed model does have certain limitations. One such limitation becomes evident when examining the resulting images presented in Figure 5 and Figure 6. It is apparent that our model faces challenges in accurately estimating target pixel values, particularly when the input image contains a significant shadowed region. This limitation arises from situations where the original images contain extensive shadowed areas, causing the model to struggle with estimating both the foreground pixels/objects and the intricate details hidden within them. Moreover, it is noteworthy that while the fusion of depth maps provides valuable front-facing spatial information, it lacks multi-directional context. As a result, although the color temperature from the input images is faithfully transferred to the output image, the model may sometimes struggle to accurately reconstruct the shadowed portion, leading to suboptimal structural fidelity.

Looking ahead, future research could focus on developing an end-to-end image restoration approach to effectively address the challenges of shadow removal and relighting. Such an approach holds the potential to further refine the capabilities and versatility of our model, pushing the boundaries of its performance in this captivating field.

## 6. Conclusions

Image relighting, a foundational process in the realm of computer vision, involves modifications in the lighting conditions while upholding the inherent visual content. In the context of this study, we introduce a bi-modal lightweight deep learning framework tailored for depth-guided relighting. Our model capitalizes on the prowess of the Res2Net Squeezed block to capture long-range dependencies and enhance the feature representation pertaining to both the input image and its corresponding depth map. The proposed model adopts an encoder–decoder structure with Res2Net Squeezed blocks integrated at each stage of encoding and decoding. The enhanced feature representation and improved information flow within the Res2Net Squeezed blocks enable the model to handle complex lighting variations and generate realistic relit images.

Thorough comparisons with previous state-of-the-art (SOTA) methods and detailed studies conducted on the innovative VIDIT dataset [6] highlight the effectiveness and efficiency of our proposed method. This is measured using metrics such as PSNR and SSIM, as well as visual quality. Our proposed approach proves to be more effective, achieving competitive performance with fewer parameters—only 0.45 million, to be precise. Furthermore, the model’s size is just 3.4 MB, making it suitable for efficient use on a range of edge devices. Taking into account the practical implementations discussed in Section 1, our proposed model holds great applications across various fields. It opens up possibilities for enhancing visual quality, realism, and user experiences in scenarios that unfold in real-time. This model provides users and professionals with enhanced control over lighting conditions, thereby boosting creativity, productivity, and precision in a variety of applications, all at a minimal cost.

## Figures and Tables

**Table 1:** Quantitative evaluation for residual strategy.

TYPE	SSIM	PSNR
w/o Residual Learning	0.6734	16.88
Vanilla Residual Block [46]	0.6801	17.34
Vanilla Res2Net [12]	0.7008	18.09
Res2Net-Squeezed block	0.7185	19.48

**Table 2:** Quantitative evaluation for coordinate convolutional layer.

TYPE	SSIM	PSNR
w/o Coordinate Conv layer	0.6878	16.88
w Coordinate Conv layer	0.7185	19.48

**Table 3:** Quantitative comparison with state-of-the-art methods.

TYPE	SSIM	PSNR	LPIPS
pix2pix [36]	0.489	15.59	0.4827
DRN [48]	0.6151	17.59	0.392
WDRN [1]	0.6442	17.46	0.3299
DPR [51]	0.6389	18.01	0.3599
OIDDR-Net [49]	0.7039	18.4	0.2837
S3Net [10]	0.7022	19.24	-
MBNet [38]	0.7175	19.36	0.2928
IAM [50]	0.7234	19.7	0.2755
**Ours**	**0.7185**	**19.48**	**0.2831**

All numbers in bold represent the results of the proposed model.

**Table 4:** Comparison of parameters and performance of state-of-the art methods.

Method	Parameters (in Millions)
pix2pix [36]	57
DRN [48]	117
WDRN [1]	8.5
DPR [51]	0.7
OIDDR-Net [49]	50
MBNet [38]	155
IAM [50]	2.67
**Ours**	**0.45**

All numbers in bold represents the results of the proposed model.

## Data Availability

Not applicable.
